# Hypoglycin A in Cow’s Milk—A Pilot Study

**DOI:** 10.3390/toxins13060381

**Published:** 2021-05-26

**Authors:** Mandy Bochnia, Jörg Ziegler, Maren Glatter, Annette Zeyner

**Affiliations:** 1 Institute of Agricultural and Nutritional Sciences, Martin Luther University Halle-Wittenberg, 06120 Halle (Saale), Germany; mandy.bochnia@landw.uni-halle.de (M.B.); maren.glatter@landw.uni-halle.de (M.G.); 2 Department of Molecular Signal Processing, Leibniz Institute of Plant Biochemistry, 06120 Halle (Saale), Germany; joerg.ziegler@ipb-halle.de

**Keywords:** hypoglycin A, raw milk, intoxication, carry-over, transfer

## Abstract

Hypoglycin A (HGA) originating from soapberry fruits (litchi, and ackee) seeds or seedlings from the sycamore maple (SM) tree (related to Sapindaceae) may cause Jamaican vomiting sickness in humans and atypical myopathy in horses and ruminants. A possible transfer into dairy cow’s milk cannot be ruled out since the literature has revealed HGA in the milk of mares and in the offal of captured deer following HGA intoxication. From a study, carried out for another purpose, bulk raw milk samples from four randomly selected dairy farms were available. The cows were pastured in the daytime. A sycamore maple tree was found on the pasture of farm No. 1 only. Bulk milk from the individual tank or milk filling station was sampled in parallels and analyzed for HGA by LC-ESI-MS/MS. Measurable concentrations of HGA occurred only in milk from farm No. 1 and amounted to 120 and 489 nmol/L. Despite low and very variable HGA concentrations, the results indicate that the ingested toxin, once eaten, is transferred into the milk. However, it is unknown how much HGA the individual cow ingested during grazing and what amount was transferred into the bulk milk samples. As a prerequisite for a possible future safety assessment, carry-over studies are needed. Furthermore, the toxins’ stability during milk processing should also be investigated as well.

## 1. Introduction

Hypoglycin A (HGA) is known as the causative agent of Jamaican vomiting sickness [1,2,3,4,5,6,7] as well as atypical myopathy in horses [8,9,10]. Fatalities in humans have been reported worldwide as a result of HGA ingestion through soapberry fruits during litchi-harvesting season with a mortality rate of > 30%; mainly young children are affected [11]. In horses, the ingestion of seeds or seedlings from the sycamore maple (SM) tree (*Acer pseudoplatanus*), which is related to the same plant family (*Sapindaceae*) as soapberry fruits, led to AM in horses with an average fatality rate of 74 % [12]. Methylenecyclopropylglycine (MCPrG), a lower homologue of HGA, was also linked to suspected cases of hypoglycemic encephalopathy, similar to Jamaican vomiting disease in humans [13] as well as to AM cases in horses [14]. In ruminants, AM cases were reported for captured David’s deer (*Elaphurus davidianus*) after ingestion of huge quantities of seeds and seedlings [15] as well as leaves and branches [16], respectively, from pruned maple trees. The presence of MCPrG and metabolites of HGA and MCPrG in the kidney and liver of dead David’s deer [16] and HGA in the muscle [17] and hair [18] of affected horses (MCPrG and its metabolites are not investigated in this study), might serve as evidence for the possibility of SM toxin transfer from feed into the tissues of both horses and ruminants. This is in line with in vitro results showing that HGA is remarkably resistant during inoculation to equine gastric [19] as well as bovine [20] and ovine rumen content [19], and thus potentially available for absorption. If SM toxins can be transferred from the feed into organs and tissues of horses and ruminants, it is reasonable to assume that they can also be transferred into their milk. Indeed, several studies report intoxications of gravid mares and their suckling foals [12]. Any diaplacental transfer of SM toxins cannot be excluded from the cited research, but foals might also be poisoned by a mare’s milk. In fact, the milk of an AM affected mare, whose foal showed signs of illness, contained HGA, MCPrG and associated metabolites [21]. In a previous study, HGA and its metabolite methylenecyclopropylacetyl-carnitine (MCPA-carnitine; MCPrG and its metabolites not investigated in this study) were measured in milk samples from three out of four grazing mares exposed to SM trees [22]. Moreover, one of six commercial mare’s milk samples contained methylenecyclopropylformyl-glycine and -carnitine. The last two are metabolites of MCPrG [21]. Ruminants can also be exposed to the ingestion of seedlings and seeds of SM trees containing HGA in spring and autumn, respectively. HGA has been detected in the blood serum of sheep (5.62–126.4 ng/mL), grazing on pastures contaminated with SM seedlings, as well as their nursing lambs (8.82–23.71 ng/mL), suggesting that HGA is excreted in milk [19]. Based on the probable resistance of HGA to ruminal degradation [19,20], and the reported concomitant occurrence of HGA in the blood serum of sheep grazing on contaminated pastures and their nursing lambs [19], we hypothesized that a transfer of ingested HGA into cow´s milk is possible, at least in principle. The aim of this pilot study was to investigate HGA occurrence in bulk cow´s milk taken from four randomly selected dairy farms keeping cows on pasture. SMs were grown on one farm pasture only. 

## 2. Results

### 2.1. HGA Analysis in Raw Milk Samples

The HGA concentrations detected in the different raw milk samples are shown in Table 1. HGA concentrations ≥ 9 µg/L were detected in both sub-samples of the milk from farm No. 1 only. 

### 2.2. Extracted Ion Chromatograms for HGA

**Figure 1:**
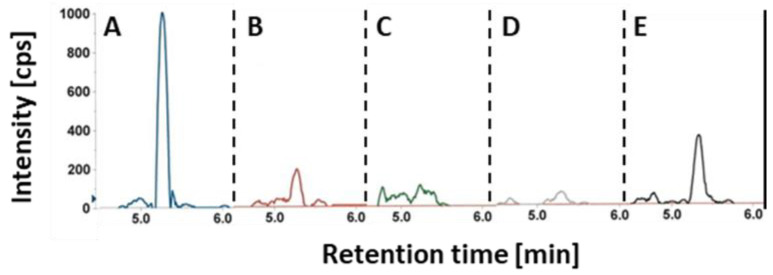
Extracted ion chromatograms (transition *m/z* 362 → *m/z* 166; [HGA-Fmoc-H]^−^ → [HGA-2H+CO-H]^−^). (**A**) sample A (blue trace) and (**B**) sample B (red trace) containing HGA; (**C**) sample A (green trace) and (**D**) a blank sample (gray trace), which do not contain HGA. (**E**) HGA standard 18 µg/L (black trace).

Besides the quantitative illustration of the HGA concentrations in Table 1, Figure 1 shows the extracted ion chromatogram for HGA contents in the milk.

## 3. Discussion

To our knowledge, this is the first study to report the presence of HGA in the milk of dairy cows. HGA was measured only in the milk of cows exposed to SM on pasture. This supports the assumption that a transfer of orally ingested SM toxin into the milk occurred. However, both sub-samples of bulk raw milk from the farm in question contained quite different concentrations of HGA (120 and 489 nmol/L). This high variation might in part be explained by an unequal distribution of HGA among individual milk fractions. Preliminary results of an ongoing study reveal a clear majority of HGA occurring in whey (data not revealed here). The sequential filling of sub-samples of milk with subsequent freezing and thawing might lead to unequal HGA concentration between individual milk fractions within the same sample. Indeed, the milk samples showed different degrees of coagulation. The procedure of sampling, homogenization, freezing and thawing of milk, as well as the production of milk serum for HGA analysis, thus needs to be optimized and standardized to guarantee repeatable results. In the milk of mares exposed to SM trees HGA concentration was 2 nmol/L (*n* = 1; [21]) and 0–9.47 (mean 4.09 ± 4.10) nmol/L (*n* = 4; [22]). Prior to analysis, the mares milk samples were extracted with a methanolic solution [21], or the pre-analytical handling was not described in detail [22]. Nevertheless, the HGA concentrations measured in cow´s milk in the present study were up to 52-fold higher than the maximum concentration measured in mare’s milk [22]. The mare’s milk also contained MCPA-carnitine [22], methylenecyclopropylformyl-carnitine and methylenecyclopropylformyl-glycine (metabolites of MCPrG), as well as elevated concentrations of several acylcarnitines, indicating the inhibition of ß-oxidation [21]. As was shown in previous studies [1,8,9], it is essential to understand whether animals ingest the toxic plant materials during grazing (e.g., from seeds or seedlings) or whether they ignore these materials. Despite cows showing a different feed intake behavior and having a different digestive tract anatomy to horses, it can also be assumed that they ingest HGA via SM seeds or seedlings while grazing. Individual cows on the same pasture might vary regarding ingested amounts of HGA. The present study did not evaluate HGA concentrations in the seeds. Several previous studies have shown a high variety of HGA in SM seeds (2–1.340 µg HGA/g) [8,10,14]. The availability of HGA and MCPrG for animals is linked to the vegetation period of SM trees, which explains AM outbreaks in horses mainly in spring (seedlings) and autumn (seed fall in the wind and rain) [23]. The additional aspect that pruning increases the HGA content in SM seedlings and may furthermore contaminate surrounding material [24] would be an important fact in the production of total mixed rations for dairy cows if cut seeds or seedlings are involved. The cut plant material and the subsequent production of silages or hay with no significant degradation of HGA [24] might lead to year-round HGA exposure. Therefore, the link to the food chain needs to be considered further, especially regarding products from lactating animals used for human consumption. Here, not only does a fast accumulation of HGA and/or MCPrG bear a risk, but so too does long-term accumulation. Additionally, cow’s milk could contain HGA and be its source. Before any eventual future safety assessment regarding food safety through milk contamination, carry-over studies under experimental conditions are needed, and investigations into toxins’ resilience during milk processing should also be conducted

## 4. Conclusions

This study indicates that HGA, once ingested, can be transferred into the milk of dairy cows. Further research is needed to assess the possible risks of HGA and other maple toxins´ exposure via milk and other dairy products pose to human beings. 

## 5. Materials and Methods

### 5.1. Farms, Cows and Milk Sampling 

Raw milk samples from four randomly selected farms in Northern Germany (Schleswig Holstein and North Friesland) were collected in spring 2019. The dairy cows from all the investigated farms had pastured during the day. At farm No. 1, an SM tree (*Acer pseudoplatanus*) was identified on the pasture. None of the cows showed clinical signs of AM. The milk was sampled directly from the milk tank on the farm or from the milk filling station which belonged to the individual farms. After sampling, the milk was frozen at −20 °C. Each sample consisted of two sub-samples (A and B), which were separately filled up for later analysis. 

### 5.2. Storage, Preparation and Analytical Procedure for HGA Detection in Milk Samples

In the lab, the milk samples were stored at −80 °C until analysis. After thawing, the raw milk samples showed strongly different degrees of coagulation and the particulate matter was precipitated by centrifugation at 12,000 g for 10 min. A total of 23 µL of milk serum (being the supernatant after centrifugation) was used for the determination of HGA by LC-ESI-MS/MS and quantified as its Fluorenylmethoxycarbonyl (Fmoc)-derivative according to the protocol for amino acid determination published by Ziegler et al. [25]. Calibration for the determination of the limit of detection was performed in triplicate by spiking several concentrations of HGA (Toronto Research Chemicals, Toronto, Canada; 0 µg/L, 2.3 µg/L, 4.5 µg/L, 9 µg/L, 18 µg/L, 36 µg/L, 146 µg/L, 587 µg/L, 2350 µg/L, 4700 µg/L) into pasteurized and homogenized milk obtained from a local supermarket. The samples were processed as described [25]. Data were summarized into a calibration curve (R^2^ = 0.96). Signals consistently higher compared to control samples which did not contain HGA, could be obtained at HGA concentrations of 9 µg/L (signal to noise ratio ~15).

## Figures and Tables

**Table 1:** HGA concentration in raw milk samples taken from milk tank or milk filling stations from different dairy farms.

Milk Sample ^1^	Fraction	HGA (µg/L)	HGA (nmol/L)
1A	raw milk	69	489
1B	raw milk	17	120
2A	raw milk	<LOD	<LOD
2B	raw milk	<LOD	<LOD
3A	raw milk	<LOD	<LOD
3B	raw milk	<LOD	<LOD
4A	raw milk	<LOD	<LOD
4B	raw milk	<LOD	<LOD

<LOD, below the lower detection limit which is 9 µg/L; ^1^ milk samples from dairy farms (no. 1–4) taken as two parallel sub-samples (A, B) at the same time point from the same vessel.

## Data Availability

All data are available in this publication.
